# Preparation, Characterization, and In Vivo Evaluation of an Oral Triptolide Nanomatrix System for Rheumatoid Arthritis Therapy

**DOI:** 10.3390/pharmaceutics17121567

**Published:** 2025-12-05

**Authors:** Yujian Liang, Mingyu Li, Qing Zhou, Chenyang Liu, Longfei Lin, Liuqing Yang, Wenbing Dai

**Affiliations:** 1 State Key Laboratory of Natural and Biomimetic Drugs, School of Pharmaceutical Sciences, Peking University, Beijing 100191, China; 2 Department of Sports Medicine, Peking University Third Hospital, Institute of Sports Medicine of Peking University, Beijing 100191, China; 3 Institute of Chinese Materia Medica, China Academy of Chinese Medical Sciences, Beijing 100700, China

**Keywords:** triptolide, solid nanomatrix, rheumatoid arthritis, dissolution behavior, Eudragit^®^

## Abstract

**Background**: Triptolide (TP), a principal bioactive component of *Tripterygium wilfordii*, exhibits potent anti-inflammatory activity. However, its application is still limited due to its poor solubility and systemic toxicity, primarily caused by uncontrolled absorption after oral administration. Our previously established oral nanomatrix system, composed mainly of commercially available nanoporous Sylysia and Eudragit^®^, can not only enhance the in vitro dissolution of poorly water-soluble drugs, but also modulate their absorption sites in gastrointestinal tract. **Methods**: We prepared a TP nanomatrix system using Sylysia 350 and Eudragit^®^ L100 to modulate TP’s dissolution in order to overcome TP’s limitation. Then, the nanomatrix was evaluated through in vitro dissolution, physicochemical characterization, and in vivo pharmacokinetic study, and then was comprehensively assessed for efficacy and safety in a rat model of rheumatoid arthritis. **Results**: TP nanomatrix system exhibited a marked increase in drug dissolution in various media, especially in pH 6.8 medium. The nanomatrix system showed better oral bioavailability than free TP, yet with no toxicity observed. **Conclusions**: This study developed a simple oral nanomatrix system of TP with enhanced anti-inflammatory efficacy without observed toxicity, and provided a potential strategy to progress the clinical translation of TP products.

## 1. Introduction

*Tripterygium wilfordii* is recognized as one of the “poster children” for the promise of turning traditional medicines into modern drugs [1]. As one of the most widely used and clinically validated treatments for rheumatoid arthritis (RA), *Tripterygium* preparations such as Tripterygium Glycosides Tablets have been successfully applied and widely studied for years [2,3,4]. The latest international consensus guidelines for RA treatment further recommend *Tripterygium* as the first-line therapeutic option and combination therapy with methotrexate [5].

Triptolide (TP), the primary bioactive and toxic component of *Tripterygium wilfordii*, has been demonstrated to have remarkable anti-inflammatory efficacy in various autoimmune diseases [6], including RA [7] and systemic lupus erythematosus [8,9], and has also been extensively investigated for anticancer applications [10,11]. However, the clinical translation of TP is hampered by its biopharmaceutical properties and safety profile. It was found that oral administration of TP exhibited a rapid absorption followed by a rapid elimination [12,13], which could cause undesired clinical outcomes. Nevertheless, as a Biopharmaceutics Classification System Class II drug, TP shows poor water solubility [14], which resulted in low bioavailability. Therefore, it is important to overcome its limitations for the clinical translation of TP.

Current research strategies of TP oral products can be grouped into three main categories: (1) combination regimens leveraging drug–drug interactions, (2) structure-based prodrug design [15], and (3) oral drug delivery systems.

Numerous studies focused on drug combinations to modulate the systemic exposure of TP by regulating TP’s metabolism via enzymes or transporters like CYP3A4 and P-gp [16,17,18,19]. Other combination strategies utilized specific phytochemicals for targeted organ protection [20,21,22]. However, the clinical translation of such complex regimens remains challenging owing to the low druggability of the extra active pharmaceutical ingredients. The primary objectives of developing oral prodrugs of TP are to enhance its water solubility, extend its half-life, and reduce its toxicity [23,24]. For instance, the conjugate of TP and chitosan oligosaccharide has been shown to significantly increase bioavailability. Nevertheless, these efforts primarily focus on improving systemic circulation and mitigating toxicity, most of which lack systematic evaluation of therapeutic effect for rheumatoid arthritis. Alternatively, several delivery technology-based oral TP formulations have been investigated. For instance, solid lipid nanoparticles, which could delay *T_max_* and reduce *C_max_*, have showed a promising toxicity reduction in rodents [12]. A double-layer tablet (containing 50 μg of TP per tablet) developed in China, with 30% immediate-release and 70% sustained-release, exhibited a lower peak concentration [25,26]. However, several cases of toxicity were still reported in clinical trials [27]. Notably, such oral delivery systems focusing on toxicity reduction often overlooked verifying whether attenuated fluctuations in plasma drug concentrations maintain or even enhance therapeutic efficacy. Another point worth noting is that, following oral administration, TP reached its peak plasma concentration (*C_max_*) within about 10 min [28]. As reported, TP predominantly existed in its molecular form under acidic conditions, which favored rapid absorption. Among all intestinal segments, the duodenum exhibited the fastest absorption rate for TP. It is therefore inferred that the primary absorption sites of TP were stomach and duodenum [29,30,31,32,33]. While some systems successfully enhanced solubility [34,35,36], they frequently overlooked the critical importance of modulating the rapid and erratic absorption profile of TP. Based on this, it is worth trying to address the toxicity issues of TP through adjusting the inherent absorption preference in stomach and intestinal tract.

In summary, existing strategies for TP oral administration either are too complex for clinical translation, or primarily focus on preparation design and characterization, lacking comprehensive evaluation of its anti-arthritis effect. It is meaningful to develop a simple and safe oral formulation for TP and systematically validate the in vivo anti-arthritis performance.

To address the problems, we leverage a simple, stable, and commercially proven oral nanomatrix platform. Unlike many exploratory nanocarriers, our system is exclusively composed of commercially available pharmaceutical excipients, Sylysia and Eudragit^®^, and a poorly soluble drug. This nanomatrix system can be prepared by a simple process and have been wildly validated to significantly enhance the solubility of multiple therapeutic agents [37,38,39,40]. Importantly, one of nanomatrix system products is in the final stage of regulatory approval in China. Functionally, it could enhance oral drug delivery through a dual mechanism. First, it could improve drug dissolution rate through highly dispersing the drug molecules, and also provide bioadhesion in the gastrointestinal tract, both of which contribute to the oral bioavailability enhancement of poorly water-soluble drugs. Second, it could modulate drug release site within the gastrointestinal tract by selecting Eudragit^®^ with different pH-sensitive solubility. Therefore, in this study, we aim to explore the feasibility of exploiting the oral nanomatrix technology to address the challenges of clinical translation of TP.

Herein, we hypothesize that applying this mature technology to TP would yield a formulation capable of not only enhancing TP’s solubility but also modulating its release site within the gastrointestinal tract, therefore enhancing the anti-arthritis efficacy of TP for RA. Based on our previous research and experience of product development, we naturally chose commercially Sylysia 350 (S350) as the solid framework and Eudragit^®^ L100 (EL100) as bioadhesive and pH-responsive polymer to control TP’s dissolution behavior [37,38,39,40]. In collagen-induced arthritis rat model, the anti-arthritis efficacy and also the side effect of this nanomatrix were systematically evaluated. This work provides a proof-of-concept validation for this platform, by comprehensively evaluating the anti-arthritis efficacy, pharmacokinetics, and safety of the TP-loaded nanomatrix. The findings offer a foundation for the future development of oral TP formulations.

## 2. Materials and Methods

### 2.1. Materials

Triptolide (38748-32-2) was obtained from Chengdu Pufei De Biotech Co., Ltd. (Chengdu, China). Eudragit^®^ L100 was provided from Evonik Industries AG (Darmstadt, Germany). Sylysia 350 (Fuji Silysia) was purchased from Guangzhou Standard Pharma Ltd. (Guangzhou, China). Collagen 2 (Chondrex, 20022) and Incomplete Freunds Adjuvant (Chondrex, 7002) were purchased from Biolead Biology Sci & Tech Co., Ltd. (Beijing, China). 4% Paraformaldehyde (P1110), stroke-physiological saline solution (HDLS001119), Masson’s Trichrome Stain Kit (G1340), Modified SO/FG Stain Kit (G1371), and Hematoxylin-Eosin Stain Kit (G1120) were purchased from Beijing Solarbio Science & Technology Co., Ltd. (Beijing, China).

Rat TNF-α ELISA Kit (ER20497M), Rat IL-6 ELISA Kit (ER20498M), and Rat IL-1β ELISA Kit (ER20275M) were purchased from Shanghai Weiao Biotechnology Co., Ltd. (Shanghai, China). Hematoxylin-eosin HD constant dye kit (G1076) was purchased from Wuhan Servicebio Technology Co., Ltd. (Wuhan, China). AST activity assay kit (SC19N0200), ALT activity assay kit (SC20N0200), UREA activity assay kit (SC28N0200), CK activity assay kit (SC32A0300), and LDH activity assay kit (SC42N0200) were purchased from Wuhan Life Origin Biotech Joint Stock Co., Ltd. (Wuhan, China). CREA activity assay kit (105-000492-00) was purchased from Shenzhen Mindray Bio-Medical Electronics Co., Ltd. (Shenzhen, China).

### 2.2. Preparation of TP Nanomatrix System

TP nanomatrix system was prepared using a solvent evaporation method. The ratio of TP:EL100:S350 was fixed at 1:5:3 (*w*/*w*/*w*) in this preliminary work. First, EL100 and TP were added into 80 mL of anhydrous ethanol, followed by ultrasonic disperse process until completely dissolved. Subsequently, the prescribed quantity of S350 was added to the solution, and then the suspension was continuously stirred for 30 min and further homogenized by an additional 30 min of sonication at room temperature (RT). The solvent was completely evaporated under reduced pressure at 40 °C using a rotary evaporator. During evaporation, the increasing solute concentration facilitated effective drug adsorption into the mesopores of S350 [41].

The resulting solid matrix was carefully scraped from the flask wall, pulverized using a mortar and pestle, sieved through a 100-mesh sieve, and stored in airtight containers under cool and dry conditions until further use.

### 2.3. Determination of Production Yield and Drug Loading

#### 2.3.1. High-Performance Liquid Chromatography Analysis

The TP content analysis was performed using a Shimadzu LC-10AT high-performance liquid chromatography (HPLC) system (Shimadzu Corp., Tokyo, Japan). The chromatographic separation was carried out on a ReproSil-Pur Basic-C18 column (250 mm × 4.60 mm, 5 μm; Dr. Maisch GmbH, Ammerbuch-Entringen, Germany) maintained at 30 °C. The mobile phase consisted of water and acetonitrile (55:45, *v*/*v*) with a flow rate of 1.0 mL/min. The eluate was detected at 218 nm.

#### 2.3.2. Production Yield

Production yield was calculated to assess the efficiency of the preparation process. The total mass of the starting materials, including S350, EL100, and TP, was recorded as *M*_input_. The total mass of the final nanomatrix product was recorded as *M*_product_. The yield was then calculated according to Equation (1):

Yield (%) = *M*_input_/*M*_product_ × 100% (1)

#### 2.3.3. Drug Loading

Drug loading was measured to quantify the amount of TP loaded within the nanomatrix. Briefly, an accurately weighed amount (*w*) of the nanomatrix was dispersed in a known volume (*V*) of ethanol. The suspension was agitated at 100 rpm for 2 h to ensure complete drug extraction. After centrifugation and appropriate dilution (*Y*), the concentration of TP (*C*) in the supernatant was determined by high-performance liquid chromatography (HPLC). The drug loading was calculated using Equation (2):

Drug Loading (%) = *Y* × *C* × *V*/*w* × 100% (2)

Three aliquots of the nanomatrix were accurately weighed following a random sampling procedure. Based on the mass ratio of the components in the formulation, the theoretical drug loading was expected to be approximately 11.1% (1/9).

### 2.4. In Vitro Dissolution Study

The nanomatrix (containing 2.6 mg of TP) was placed into 20 mL of dissolution media (water, pH 1.2 hydrochloric acid solution, pH 4.5 phosphate buffer, and pH 6.8 phosphate buffer) to evaluate the pH-dependent release profile of the formulation. The mixture was incubated at 37 °C with a shake speed of 100 rpm for 60 min. At predetermined time points (5, 10, 15, 30, 45, and 60 min), an aliquot of 0.5 mL sample was collected. And an equal volume of fresh dissolution medium was added immediately. Each sample was centrifuged at 8000 rpm for 3 min, and then the supernatant was collected. The concentration of TP was determined using HPLC.

### 2.5. Physicochemical Characterization

The X-ray diffraction (XRD) patterns of the samples were analyzed using SmartLab 9 kW X-ray diffractometer (Rigaku Corp., Tokyo, Japan) equipped with a Cu Kα radiation source. Diffraction patterns were scanned over the 2θ range of 5–60° using a 5°/min scan speed [42].

The differential scanning calorimetry (DSC) of the samples was analyzed in an open aluminum sample pan using a DSC 3500 Sirius differential scanning calorimeter (Netzsch Ltd., Selb, Germany) under a nitrogen flow, heated from 30 °C to 250 °C at a rate of 10 °C/min.

The surface morphology of TP, nanomatrix, EL100, and S350 was characterized using a JSM-7900F scanning electron microscope (JEOL Ltd., Tokyo, Japan) operated at 3 kV. The samples were evenly dispersed onto a specimen stub and coated with a 3 nm thick layer of platinum before imaging.

### 2.6. Stability Study

The nanomatrix system was sealed in microcentrifuge tube and stored at RT for six months. Its morphology, crystallinity, and drug release profile were assessed at predetermined time points.

### 2.7. In Vivo Pharmacokinetics Study

#### 2.7.1. Design and Sampling

7-week-old male Sprague-Dawley (SD) rats were randomly divided into two groups. Following overnight fasting, the rats were administered either TP suspension or TP nanomatrix suspension via oral gavage at a dose of 1 mg/kg (based on TP content). Blood samples were collected at 3, 6, 10, 15, 30, 45, 60, 120, and 240 min after administration. The samples were centrifuged at 5000 rpm for 10 min, and 80 μL of plasma was collected. 80 μL of carbamazepine solution in ethyl acetate (internal standard, IS, 10 ng/mL) and 740 μL of ethyl acetate were added into plasma. After vortex for 10 min, the mixture was centrifuged. The supernatant was transferred and evaporated to dryness. The residue was dissolved in 80 μL of acetonitrile, followed by centrifugation. The resulting supernatant was collected for quantification of TP concentration using liquid chromatography-tandem mass spectrometry (LC-MS/MS).

#### 2.7.2. LC-MS/MS Analysis

LC–MS/MS analysis was performed on a Waters ACQUITY UPLC CSH C18 column (130 Å, 1.7 μm, 2.1 mm × 50 mm). The system consisted of a Waters ACQUITY Premier HPLC system (Waters Corp., Milford, MA, USA) coupled with a Waters Xevo TQ-Absolute triple quadrupole mass spectrometer (Waters Corp., Milford, MA, USA). The mobile phase comprised (A) 0.4% formic acid in distilled water and (B) acetonitrile, with the following gradient program: 0–2 min, 35–100% A; 2–3 min, 100% A; and 3–4 min, 35% A. The sample injection volume was 2 μL, and the analysis was conducted in multiple reaction monitoring (MRM) mode. The fragmentation patterns were as follows: TP: *m*/*z* 361.12 → 145.00, *m*/*z* 361.12 → 128.08, and *m*/*z* 361.12 → 115.07; IS (carbamazepine): *m*/*z* 237.13 → 179.08 and *m*/*z* 237.13 → 194.62. The retention times were 1.31 min for TP and 1.63 min for IS.

### 2.8. Construction of Collagen-Induced Arthritis Rats

Male SD rats were provided by Department of Laboratory Animal Science of Peking University Health Science Center and acclimated at 25 °C and 55% of humidity under natural light/dark conditions for one week before studies. The animal study protocol was approved by the Institutional Animal Care and Use Committee (IACUC) of Peking University Health Science Center (Protocol Code: DLASBE0025). All care and handling of animals were performed with the approval of the IACUC of Peking University Health Science Center.

To establish collagen-induced arthritis (CIA) model, 6-week-old male SD rats were allowed to acclimatize for 1 week. Using a homogenizer, the Incomplete Freund’s Adjuvant and bovine type II collagen (2 mg/mL^−1^) were mixed into high-quality emulsion, which was used for immunization. Rats were immunized by subcutaneous injection with 200 µL of the emulsion at the base of tail on day 0. On day 7, 100 µL of the emulsion was injected subcutaneously at the base of tail for booster immunization [43].

### 2.9. In Vivo Pharmacodynamics Study

#### 2.9.1. Experimental Protocols and Evaluation

On day 0, rats were randomly assigned into groups according to our findings (n = 9, in case arthritis incidence ≠ 100%). The normal control group did not receive CIA induction. The other rats in the CIA-induced group received daily oral administration from day 6 to day 42, and were euthanized on day 42. All drugs administered were dispersed in a 0.5% (*w*/*v*) sodium carboxymethyl cellulose (CMC-Na) aqueous solution. The experimental groups were: (1) control group (0.5% CMC-Na), (2) model group (0.5% CMC-Na), (3) TP-suspension group (free TP, 500 µg/kg), and (4) Nanomatrix group (TP nanomatrix, 500 µg/kg TP-equivalent).

The thickness of both hind paws was measured and averaged every 3 days. Gait analysis training and data collection were performed in rats between days 38 and 42 post-administration. On day 42, hematological and biochemical parameters were assessed at the end of the experiment. Blood was collected and centrifugated at 5000 rpm, 4 °C for 10 min for serum. TNF-α, IL-6, and IL-1β level in serum were analyzed using enzyme-linked immunosorbent assays (ELISA). Following euthanasia, ankle joints were harvested and fixed in 4% paraformaldehyde for micro-CT imaging and histological examination (H&E, SO/FG, and Masson staining). All the slides were scanned using Zeiss axioscan 7 (Carl Zeiss Microscopy GmbH, Jena, Germany).

#### 2.9.2. Arthritis Incidence

Arthritis incidence was systematically assessed by clinical evaluation of joint swelling and erythema [44]. When hind paws of rats were swelling and the thickness of the hind paws were increased in subsequent measurements, rats were classified into the arthritis-positive group for incidence calculations.

#### 2.9.3. Motion Gait Analysis

To evaluate arthritis-induced locomotor impairment, quantitative gait assessment was undertaken according to the manufacturer’s protocol. Following a 4-day acclimation period with daily training sessions to reduce stress-induced gait artifacts, animals walked voluntarily across an enclosed walkway (MAG, Shanghai Yuyan Scientific Instruments Co., Ltd., Shanghai, China). Gait parameters were recorded using a high-speed camera (GH5, 1080P, 50 fps) positioned perpendicular to the walkway. All acquired gait data were analyzed using the specialized motion capture software Motion Gait 1.0.

#### 2.9.4. Micro-CT Analysis

Ankle joints of rats (n = 6 per group) were scanned using a high-resolution micro-CT system (Bruker Skyscan 1276, Kontich, Belgium) with the following acquisition parameters: 0.25 mm aluminum filter for beam hardening reduction; 360°rotation with 450 steps (0.8° increment); Voltage: 50 kV, current: 200 µA; Exposure time: 1000 ms/projection; Pixel binning: 2× (effective isotropic resolution: 20 µm). Bone mineral density was calibrated using a hydroxyapatite phantom (0.3–1.25 g/cm^3^ density range) scanned under identical conditions.

### 2.10. Safety Evaluation

Body weight of the rats in each group was monitored throughout the experimental period. On day 42, whole blood and serum samples were collected. Whole blood was subjected to complete blood count (CBC) analysis, while serum was used for biochemistry analysis. Following euthanasia, major organs (heart, liver, spleen, lung, and kidney) were collected for H&E staining to assess systemic toxicity.

#### 2.10.1. Biochemistry Analysis

Serum biochemical parameters, including alanine aminotransferase (ALT), aspartate aminotransferase (AST), creatinine (CREA), urea (BUN), creatine kinase (CK), and lactate dehydrogenase (LDH) were assessed using Mindray BS-370E automated clinical chemistry analyzer (Mindray, Shenzhen, China) with corresponding commercial kits according to manufacturer’s standard protocols.

#### 2.10.2. Complete Blood Count Analysis

CBC analysis was performed using a Mindray BC-5000Vet automatic hematology analyzer (Mindray, Shenzhen, China) with its matched commercial reagents according to the manufacturer’s instructions.

### 2.11. Statistical Analysis

All data were presented as Mean ± SD. The statistical comparisons were performed using an analysis of variance (ANOVA) test. A value of *p* less than 0.05 was considered to be significant.

## 3. Results and Discussion

### 3.1. Preparation of TP-NM_EL100_

**Figure 1:**
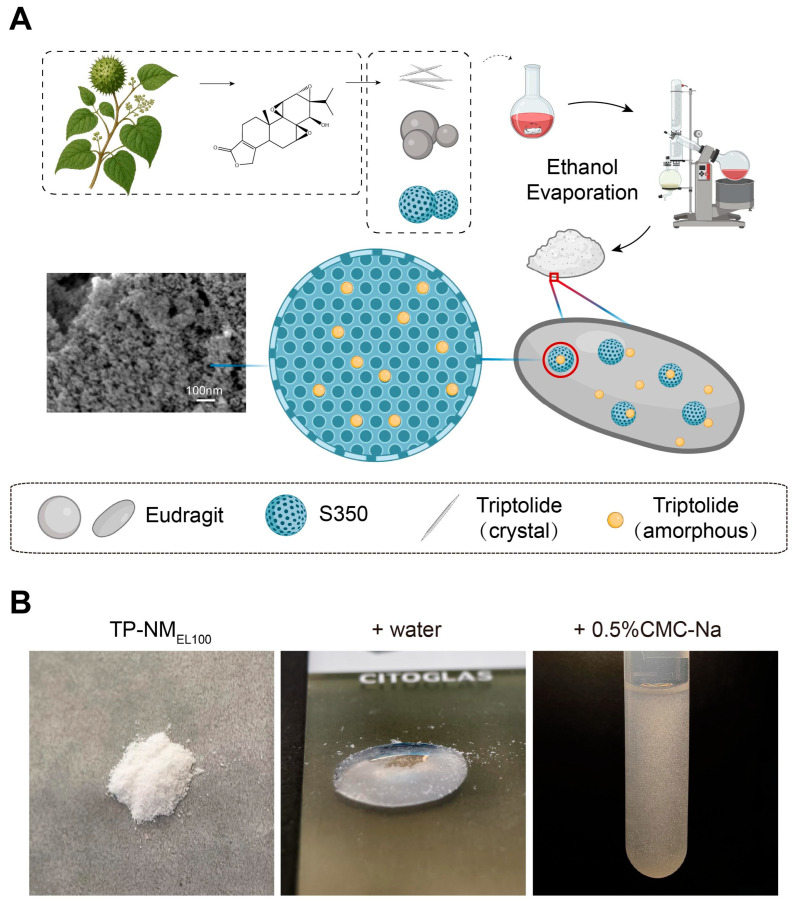
Preparation and appearance of the TP-NM_EL100_. (**A**) Preparation process of TP-NM_EL100_. Created in BioRender. Liang, Y. (2025) https://BioRender.com/mfletje. (**B**) Powder appearance, hydrophilicity and dispersibility in 0.5% CMC-Na of TP-NM_EL100_.

Mesoporous S350, EL100 and TP were utilized to prepare oral solid nanomatrix of TP. S350 was selected due to its high porosity and large specific surface area, which are critical for achieving a high drug-loading capacity and high dispersion. EL100, which dissolved at pH > 6, was chose to modulate drug release behavior in the gastrointestinal tract. The nanomatrix was successfully prepared with a rotary evaporation method (Figure 1A), and was hereafter named as TP-NM_EL100_. The TP-NM_EL100_ obtained were white powders. It displayed pronounced hydrophilicity and dispersed uniform in 0.5% CMC-Na (Figure 1B).

The preparation yield was 92.43% ± 4.56% (Mean ± SD, n = 3), calculated based on the total input mass. The drug loading of TP-NM_EL100_ was 11.18% ± 0.14%, which was closely aligned with the theoretical value. This agreement, confirmed through random sampling, indicated a homogeneous distribution of TP within the nanomatrix. Furthermore, the particle size of the final nanomatrix powder (Figure S1), as determined by optical microscopy analysis of 150 random particles, was 168.8 μm ± 73.0 μm (Mean ± SD).

### 3.2. Characterization of TP-NM_EL100_

**Figure 2:**
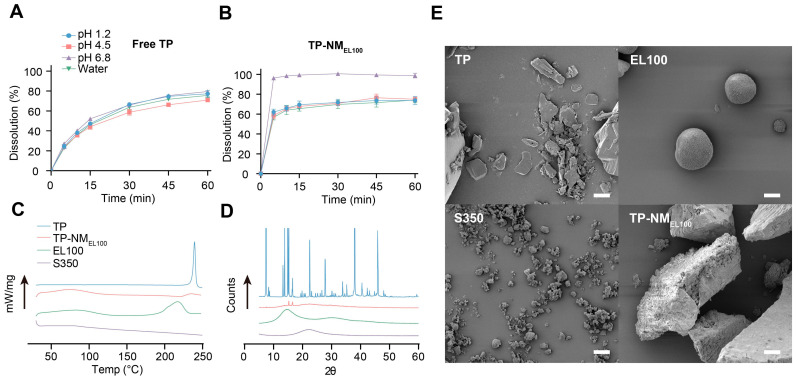
Characterization of TP-NM_EL100_ and its components. (**A**,**B**) Dissolution profiles of free TP (**A**), and TP-NM_EL100_ (**B**) in water and buffer solutions at pH 1.2, 4.5, and 6.8 (Mean ± SD, n = 3). (**C**–**E**) DSC thermograms (**C**), X-ray diffractograms (**D**) and SEM images (**E**) of TP, EL100, S350, and TP-NM_EL100_ (scale bar: 10 μm).

Next, the dissolution behaviors of free TP and TP-NM_EL100_ were evaluated in various media. It was found that free TP dissolved slowly and almost equally in all media, specifically, about 20% at 5 min and 70% at 1 h (Figure 2A). TP in TP-NM_EL100_ dissolved rapidly at pH 6.8, achieving complete TP release within 1 h. In other media, TP was released from TP-NM_EL100_ faster within the first 15 min, which can be attributed to the enhanced dispersion. However, the cumulative release at 1 h reached about 60%, which was similar to free TP under the same condition, likely due to the pH-responsive property of EL100, which dissolved at pH above 6 (Figure 2B). Therefore, TP-NM_EL100_ significantly enhanced the in vitro dissolution rate of TP in various medium. Furthermore, TP-NM_EL100_ modulated the drug release profile of TP in physiological environment-related medium to some extent, providing the base of modulating in vivo absorption behavior.

The dissolution behaviors of TP-NM_EL100_ in simulated gastric and intestinal fluids were also evaluated. A similar pH-responsive behavior was found, which further confirmed its suitability for in vivo studies (Figure S2). This pH-responsive release profile, which restructured TP’s original non-selective dissolution behavior, represented a key innovation of our nanomatrix system.

Subsequently, the characterization of TP-NM_EL100_ was further conducted. The DSC thermogram of free TP displayed a sharp endothermic peak at 239.5 °C (Figure 2C), which were not found in that of EL100, S350, and TP-NM_EL100_. It was indicated that TP was dispersed in TP-NM_EL100_ in a molecular or amorphous form.

The X-ray diffractogram of free TP exhibited several strong and sharp characteristic diffraction peaks, indicating that free TP existed in a crystalline form. There were no such characteristic peaks for EL100 and S350. The characteristic peaks of TP completely disappeared (Figure 2D), further confirming its amorphous state within TP-NM_EL100_, which was consistent with the DSC analysis results.

The morphology of TP-NM_EL100_ and its components were also examined. Free TP exhibited an irregular block-like morphology (Figure 2E). EL100 predominantly exhibited a rough-surfaced, spherical shape, and S350 consisted of micron-sized particles with some clusters. TP-NM_EL100_ exhibited a coarse, aggregated morphology with large particle sizes. It was suggested that the EL100 formed a continuous network in which the S350 particles as well as TP were embedded within and on the surface (Figure 2E).

### 3.3. Stability Study

**Figure 3:**
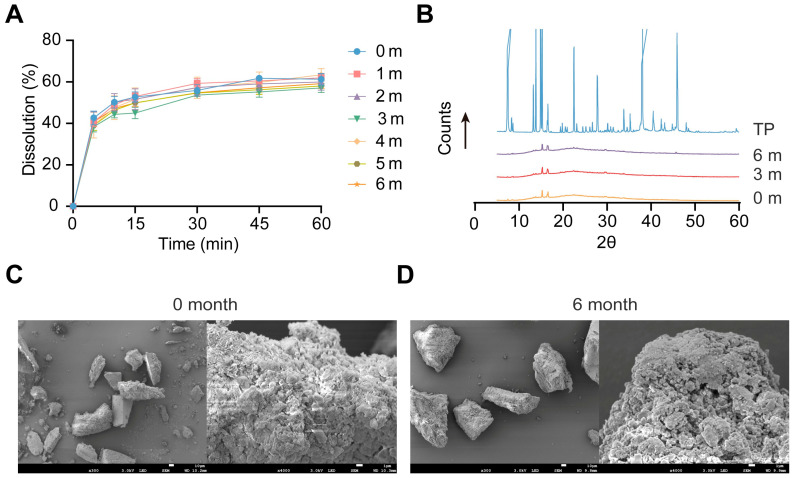
Stability of TP-NM_EL100_. (**A**) Dissolution profiles of TP-NM_EL100_ in water after storage for 0 to 6 months at RT (Mean ± SD, n = 3). (**B**) X-ray diffractograms of TP-NM_EL100_ after storage of 0, 3 and 6 months at RT. (**C**,**D**) SEM images of TP-NM_EL100_ after storage of 0 and 6 months at RT. (**left**) a representative overview (scale bar: 10 μm); (**right**) a magnified view (scale bar: 1 μm).

The stability of TP-NM_EL100_ was evaluated during storage at RT. The dissolution profiles of TP-NM_EL100_ were monthly measured, and no significant change was observed in the dissolution profiles after 6 months of storage (Figure 3A). Likewise, the X-ray diffractogram (Figure 3B) exhibited no characteristic crystalline peaks of TP, and the SEM images (Figure 3C,D) revealed no notable morphological alterations. These results suggested that TP-NM_EL100_ maintained its stability in terms of dissolution behavior, crystallinity, and structural morphology after 6 months of storage at RT. According to previous research, the drug loaded into mesoporous silica tended to undergo a transformation from the amorphous to the crystalline state during storage [45]. Our nanomatrix system maintains a remarkable long-term storage stability, which is very important for the clinical translation of TP.

### 3.4. In Vivo Pharmacokinetics Study

**Figure 4:**
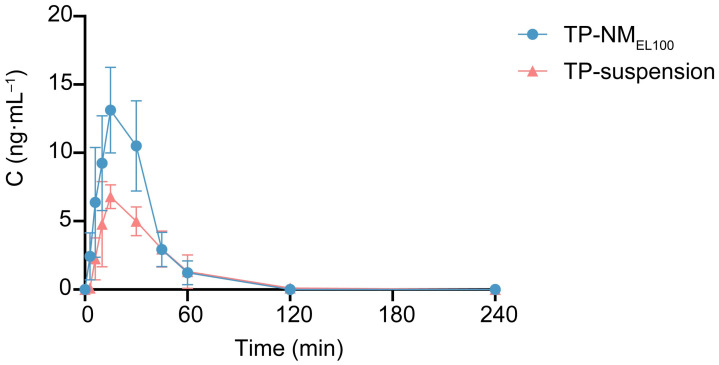
Pharmacokinetic profiles of TP-NM_EL100_. Plasma concentration–time profiles of TP in male SD rats (7-week-old) after oral administration of free TP and TP-NM_EL100_ (Mean ± SD, n = 6).

Subsequently, the pharmacokinetic study of TP-NM_EL100_ was conducted (Figure 4 and Table 1). The plasma concentration reached a peak value of only 7.54 ng/mL post oral administration of free TP, with a *T_max_* of about 15 min. The *C_max_* and AUC values of TP-NM_EL100_ were significantly higher than free TP, reaching 13.91 ng/mL and 412.28 min∙ng/mL, respectively. These values represented an approximate 84% increase in *C_max_* and a 65.6% increase in AUC compared to free TP, indicating that the loading TP into a nanomatrix markedly enhanced the oral absorption of TP in rats.

It is noteworthy that *T_max_* of TP-NM_EL100_ group showed no significant change compared to that of TP-suspension group, which was inherently related to the properties of TP. As evidenced by the in vitro dissolution results, TP was rapidly released from the nanomatrix after oral administration, with a greater amount dissolved within the first 15 min than that of the free TP group. Consequently, during this critical absorption phase for peak concentration, TP-NM_EL100_ exhibited a higher *C_max_*. Moreover, the poor dissolution characteristics of the free TP group led to its low bioavailability. In contrast, TP-NM_EL100_ released approximately 60% of its TP payload under acidic conditions, with the remainder expected to be absorbed in the subsequent intestinal segments. Unlike many reported nano-formulations that pursued maximum solubility enhancement (often leading to 2.5 to 4-fold AUC increases), our system achieved a more moderate yet safer 1.6-fold enhancement. The distinct in vivo performance of TP-NM_EL100_ underlay its improved efficacy and safety profile observed in the pharmacodynamic study.

This finding further implied that by optimizing the formulation of the nanomatrix to modulate the in vitro release profile, such as adjusting the proportion of immediate release under acidic conditions, the drug’s release and subsequent absorption behavior in the gastrointestinal tract could be altered accordingly. This rational design is highly meaningful and constitutes a key focus of our subsequent research.

### 3.5. Anti-Arthritis Efficacy of TP-NM_EL100_

**Figure 5:**
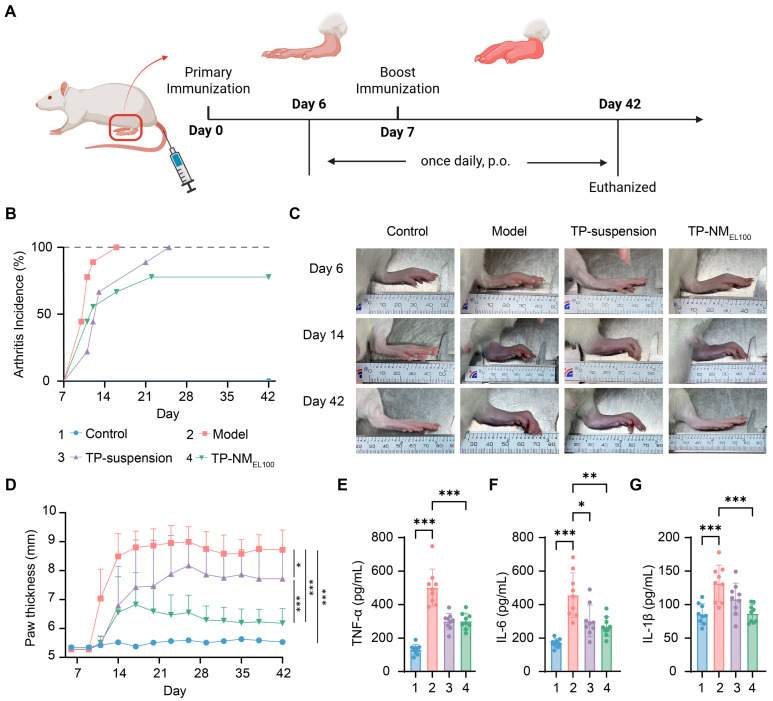
TP-NM_EL100_ enhanced the anti-arthritis efficacy of TP. (**A**) Schematic illustration of RA induction, treatment schedule, and experimental timepoints. Created in BioRender. Liang, Y. (2025) https://BioRender.com/5ld0pl0. (**B**) Arthritis incidence rates in different groups. (**C**) Representative photographs of joint morphology and erythema in each group. (**D**) Paw edema development over time. Statistical comparisons, indicated by the vertical lines on the right, were performed using the data from day 42. (**E**–**G**) TNF-α, IL-6, and IL-1β levels in plasma via ELISA. *** *p* < 0.001, ** *p* < 0.01, * *p* < 0.05 were considered statistically significant (Mean ± SD, n = 9).

Next, the in vivo anti-arthritis efficacy of TP-NM_EL100_ was evaluated. To evaluate the therapeutic potential of TP-NM_EL100_, CIA model rats were established via collagen and incomplete Freund’s Adjuvant induction (Figure 5A). Previous studies have shown that this model develops cell-mediated immune responses by day 6 after initial immunization. Thus the first administration was performed on day 6 [46], followed by daily oral gavage until the experimental endpoint (Figure 5A).

Generally, arthritis symptoms (e.g., increased paw thickness) begin to appear sporadically three days after the booster immunization. This parameter was therefore adopted to evaluate arthritis incidence. Rats in the model group rapidly developed swelling, reaching 100% incidence by day 15. In contrast, both the TP-suspension and TP-NM_EL100_ groups showed delayed arthritis onset, with some rats in TP-NM_EL100_ group remaining swelling-free until endpoint (Figure 5B), which indicated excellent anti-arthritis efficacy of TP-NM_EL100_.

In the measurement of hind paw thickness across different groups, it was observed that the paw thickness in the model group increased rapidly and remained at a high level. The TP-suspension group initially showed a mitigating effect on this process, but after 20 days, the paw thickness still increased significantly. In contrast, paw thickness of rats in TP-NM_EL100_ group exhibited noticeable decrease and maintained at a lower level after treatment (Figure 5C,D). The paw thickness statistics on day 42 further indicated that the TP-NM_EL100_ group showed the best therapeutic efficacy (Figure 5D).

This superior physiological recovery was consistent with the molecular evidence. The key inflammatory factors (TNF-α, IL-6 and IL-1β) of rats in the TP-NM_EL100_ group were significantly inhibited than that of model group. Notably, while rats in TP-suspension group also showed a significant reduction in these inflammatory markers, it was insufficient to effectively mitigate the structural joint pathology (Figure 5E–G).

**Figure 6:**
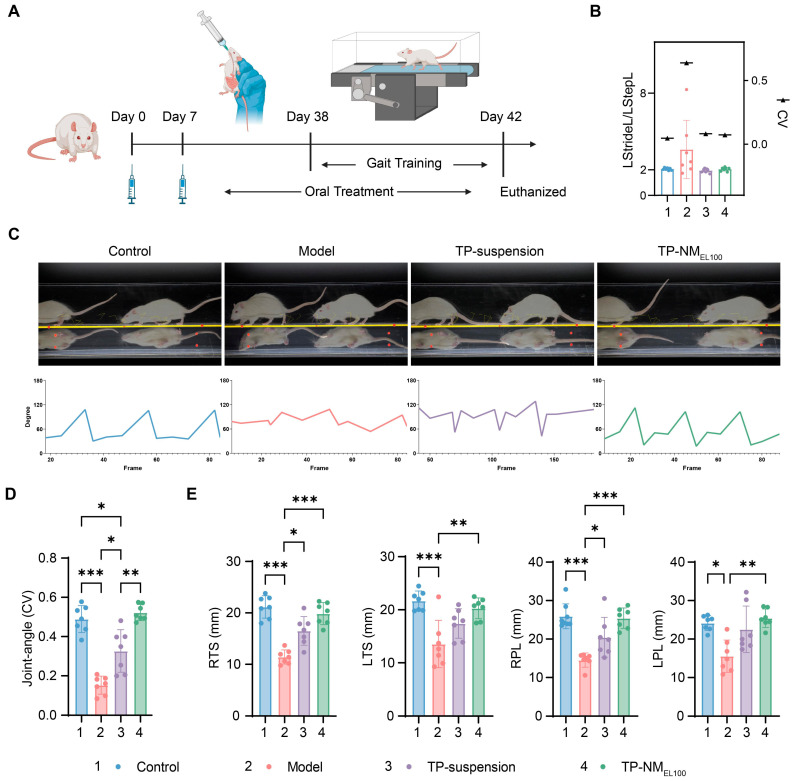
Gait analysis showing significant recovery of rats in TP-NM_EL100_ group in locomotor performance and joint function. (**A**) Schematic illustration of gait training. Created in BioRender. Liang, Y. (2025) https://BioRender.com/b4f25lm. (**B**) Stride-to-step length ratio (LStrideL/LStepL) of rats in each group, with black triangle showing within-group coefficients of variation (CV). (**C**) Representative lateral-view gait image (**top**) and representative temporal joint angle profiles (**bottom**) during locomotion. (**D**) Comparative analysis of the coefficient of variation in the ankle joint flexion-extension angle across groups. (**E**) Digitally quantified toe-spread parameters. *** *p* < 0.001, ** *p* < 0.01, * *p* < 0.05 were considered statistically significant (Mean ± SD, n = 7).

As reported, RA induces joint swelling, reduces mobility, and markedly impairs patients’ quality of life [47]. Therefore, it is essential to confirm the recovery of functional mobility. Subsequently, gait analysis was conducted and the ankle joint flexion and extension capabilities of the rats were then evaluated (Figure 6A).

Specifically, LstrideL refers to the distance between successive footfalls of the left hind paw, while LstepL is the step distance of the left hind paw relative to the right hind paw. Under normal locomotor conditions, LstrideL should be approximately twice the value of LstepL. It was showed that in the control group, this ratio was close to 2. The TP-suspension group and TP-NM_EL100_ group exhibited similar effects, maintaining this ratio. However, the model group deviated significantly from this ratio (Figure 6B). Using the coefficients of variation (CV) to assess the degree of fluctuation and found that both the TP-suspension and TP-NM_EL100_ groups could effectively restore the balance and coordination in the rats (Figure 6B).

Further analysis of gait-related parameters was performed. Generally, during a gait cycle, the hind paw ankle joint undergoes regular angular changes. However, in rats with swollen ankle joints, this movement is essentially lost (Figure 6C). Using the CV to assess this metric, it was found that rats in the model group almost completely lost their joint flexion and extension capabilities, with an average of 0.1524. In contrast, the joint angle changes in both the TP-suspension and TP-NM_EL100_ groups were significantly more periodic. Notably, the TP-NM_EL100_ group recovered to a level comparable to the control group and was significantly superior to the TP-suspension group (60%, *p* = 0.0036) (Figure 6D). The paw measurements including right forepaw stance time (RTS), left forepaw stance time (LTS), right forepaw length (RPL), and left forepaw length (LPL) further indicated that rats in the model group had a significant decrease in the area of paw contact with the ground. Although the TP-suspension group showed a little alleviation, the TP-NM_EL100_ group exhibited the best recovery (Figure 6E), indicating its superior ability in restring mobility in CIA model rats.

Taken together, the improved gait parameters likely reflected a reduction in pain and functional joint disability, a downstream consequence of the more potent systemic anti-inflammatory effect enabled by the higher drug exposure from our nanomatrix.

**Figure 7:**
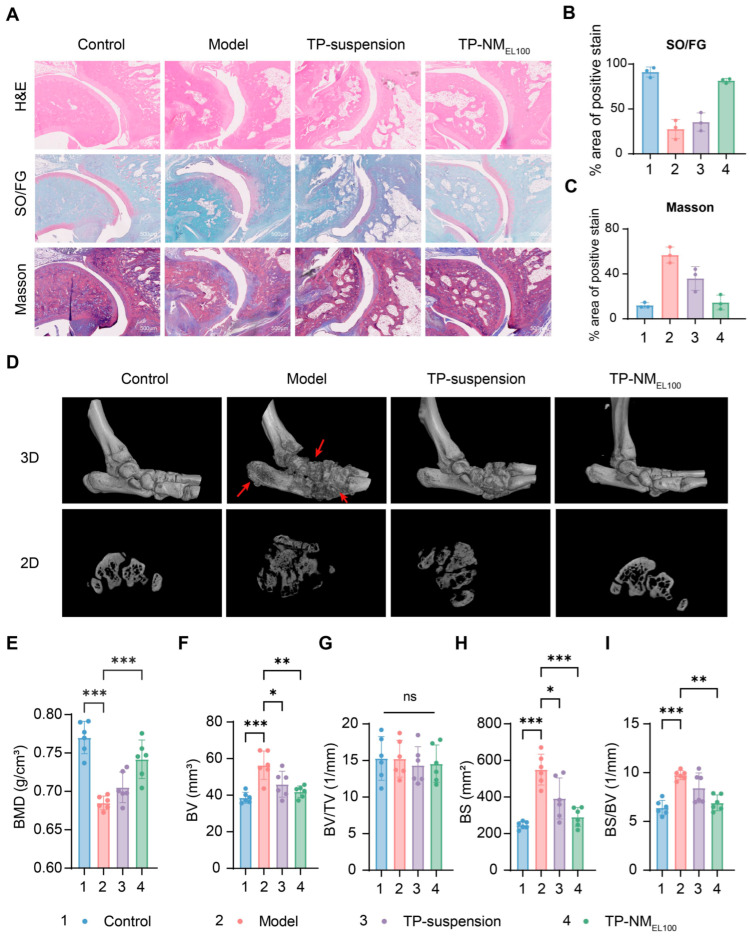
Histopathological and micro-CT analysis of ankle joints. (**A**) Representative histological sections showing H&E staining (**top**), SO/FG staining (**middle**), and Masson’s trichrome staining (**bottom**) of ankle joints (scale bar: 500 µm). (**B**) Quantification of SO/FG-positive osteoid areas. (**C**) Quantification of collagen deposition by Masson’s trichrome staining. (**D**) Images of 3D micro-CT reconstructions (**top**) and 2D micro-CT slices (**bottom**) (red arrows indicated erosive lesions). (**E**–**I**) Bone parameters of rats in different groups: (**E**) Bone mineral density (BMD), (**F**) Bone volume (BV), (**G**) Bone volume fraction (BV/TV), (**H**) Bone surface (BS), and (**I**) Bone surface density (BS/BV). *** *p* < 0.001, ** *p* < 0.01, * *p* < 0.05 were considered statistically significant (Mean ± SD, n = 3 for staining quantification; n = 6 for micro-CT analysis).

On day 42, ankle joints were collected for histological sectioning and staining (Figure 7A and Figure S3). As shown in image of Hematoxylin and eosin (H&E) staining, Safranin-O Fast Green (SOFG) staining, and Masson’s trichrome staining, the articular surfaces of rats in the model group exhibited significant inflammatory cell infiltration, severe cartilage loss, incomplete collagen layers, and extensive fibrosis. While TP-suspension group showed mild alleviation, TP-NM_EL100_ group exhibited significant improvements. These results were confirmed by quantitative staining analysis (Figure 7B,C), indicating that the nanomatrix system ameliorated joint tissue damage and promoted clinically meaningful recovery.

Ankle joints were further analyzed via micro-CT scanning and three-dimensional reconstruction. The results showed that the bone surface of normal rats was complete and smooth. In comparison, the model group exhibited severe bone erosion, which was most effectively mitigated in the TP-NM_EL100_ group (Figure 7D). This pathological pattern suggested progressive bone erosion with consequent increases in specific surface area due to trabecular perforation and microarchitecture deterioration. Quantitative analyses of bone mineral density (BMD), bone surface area (BS), and the ratio of bone surface area to bone volume (BS/BV) also supported these findings (Figure 7E–I). Compared with the model group, rats in TP-NM_EL100_ group exhibited a significant increase in BMD (8.3%, *p* < 0.001) and decrease in both BV (26%, *p* = 0.0012) and BS (47%, *p* = 0.0001). Notably, rats in TP-NM_EL100_ group showed complete preservation of bone parameters, with values remaining comparable to the control group. These mitigation of bone and cartilage damage could be attributed to the more effective suppression of the systemic inflammatory cascade, which was driven by the increased plasma concentrations of TP.

In summary, the superior therapeutic outcomes observed in TP-NM_EL100_ group, including the most significant reversal of paw swelling, improvement in gait function, and reduction in bone erosion and synovitis, were consistent with its markedly improved pharmacokinetic profile. The significantly higher AUC (Figure 4 and Table 1) indicated enhanced systemic exposure, which was crucial for exerting a potent effect in this systemic autoimmune model. These findings, which were rarely assessed comprehensively in previous studies, hold significant clinical relevance.

Moreover, it is important to note that this study correlated systemic PK with overall efficacy. Future investigations quantifying local joint drug concentrations could provide even deeper insights into the site-specific pharmacodynamics of the formulation.

### 3.6. Safety Evaluation

**Figure 8:**
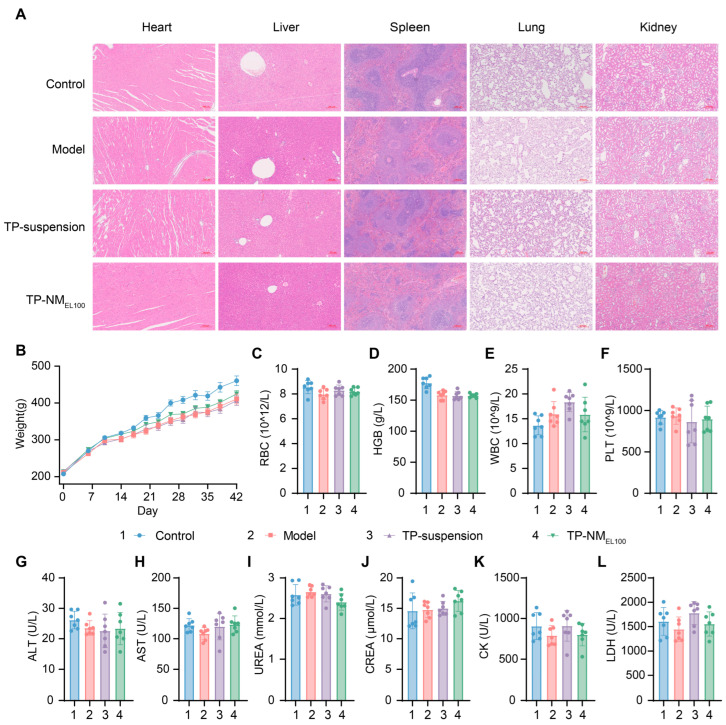
Safety evaluation of the treatment. (**A**) Representative H&E-stained sections of major organs (heart, liver, spleen, lung, and kidney) showing preserved tissue architecture across all groups (scale bar: 200 µm). (**B**) Body weight changes in rats after treatment as schedule in Figure 5A (n = 9). (**C**–**F**) Complete blood count analysis including red blood cells (RBC), hemoglobin (HGB), white blood cells (WBC), and platelets (PLT). (**G**–**L**) Serum biochemical analysis of (**G**–**H**) hepatic enzymes (ALT, AST), (**I**,**J**) renal function parameters (BUN, CREA), and (**K**,**L**) cardiac function markers (LDH, CK) (Mean ± SD, n = 7).

Along with the confirmed therapeutic efficacy of TP-NM_EL100_, it is essential to validate the safety of this approach. Toxicity of TP such as hepatotoxicity and nephrotoxicity in SD rats had been reported in previous study [6,48,49]. Subsequently, a comprehensive safety evaluation of these rats after treatment was conducted (Figure 8).

Due to the autoimmune response triggered by the emulsion, the model rats exhibited slower weight gain compared to the control group. However, no significant weight loss was observed, suggesting that the modeling process did not cause obvious damage to the rats (Figure 8B). This conclusion can be extended to the other treatment groups, implying that the rats maintained normal feeding capacity.

H&E staining revealed that there was no evident lymphocyte infiltration or abnormal cellular morphological in the hearts, livers, spleens, lungs, and kidneys of rats in all groups (Figure 8A). There was no abnormal reduction in blood cells in treatment groups, indicating favorable hematological safety (Figure 8C–F and Figure S4). Notably, although white blood cell was slightly elevated in the model group and some other groups, this increase could be attributable to the systemic inflammation elicited by CIA model induction but not considered drug-related (Figure 8E). The results of serum biochemical analyses further confirmed that key cardiac, hepatic, and renal indices remained within normal ranges (Figure 8G–L).

Multiple lines of evidence confirmed the safety of TP-NM_EL100_ in SD rats. These data indicated that our formulation could enhance the bioavailability of TP at an equivalent dosage, with no significant toxicity observed, suggesting that lower doses of TP-NM_EL100_ could achieve comparable therapeutic effects to conventional formulations in clinical settings. Such reduction in therapeutic dose would also lower the risks of cumulative toxicity.

## 4. Conclusions

In this study, we used well-established oral nanomatrix technology to prepare a novel formulation for TP to modulate the absorption behavior. The TP nanomatrix system is easily prepared with commercially available excipients. Apart from having excellent anti-arthritis efficacy, it has excellent stability and exciting biosafety. Considering there might be room for improving the formulation of TP nanomarix, the oral nanomatrix technology presents a promising candidate for the clinical translation of TP in rheumatoid arthritis therapy.

## Figures and Tables

**Table 1:** Pharmacokinetic parameters of TP after rat oral administration (n = 6).

Oral Administration	*T_max_* (min)	*T*_1/2_ (min)	*C_max_* (ng/mL)	AUC_0–240_ (min∙ng/mL)
TP-suspension	15.83 ± 7.36	25.43 ± 12.49	7.54 ± 4.45	249.03 ± 52.37
TP-NM_EL100_	16.00 ± 7.75	20.68 ± 19.86	13.91 ± 2.67	412.28 ± 109.20

## Data Availability

The original contributions presented in this study are included in the article/Supplementary Material. Further inquiries can be directed to the corresponding authors.

## Supplementary Materials

The following supporting information can be downloaded at: https://www.mdpi.com/article/10.3390/pharmaceutics17121567/s1, Figure S1: Particle size distribution of the powder of TP-NMEL100 (n = 150). Figure S2: In vitro release behavior of TP-NMEL100 in simulated gastric and intestinal fluids (Mean ± SD, n = 3). Figure S3: Representative H&E-stained joint sections from each group of rats. Figure S4: Four additional parameters of complete blood count analysis (Mean ± SD, n = 7).

## Abbreviations

The following abbreviations are used in this manuscript:

TP	Triptolide
CMC-Na	Sodium Carboxymethyl Cellulose
SEM	Scanning Electron Microscopy
DSC	Differential Scanning Calorimetry
XRD	X-Ray Diffraction
Micro-CT	Micro-Computed Tomography
