# Supplementary material for: Preparation, Characterization, and In Vivo Evaluation of an Oral Triptolide Nanomatrix System for Rheumatoid Arthritis Therapy
Source: Pharmaceutics. 2025 Dec 5;17(12):1567. doi: 10.3390/pharmaceutics17121567 (PMC12737110; doi:10.3390/pharmaceutics17121567)
Supplement: Supplementary file 1 [file pharmaceutics-17-01567-s001.zip › pharmaceutics-3982474-supplementary.pdf]

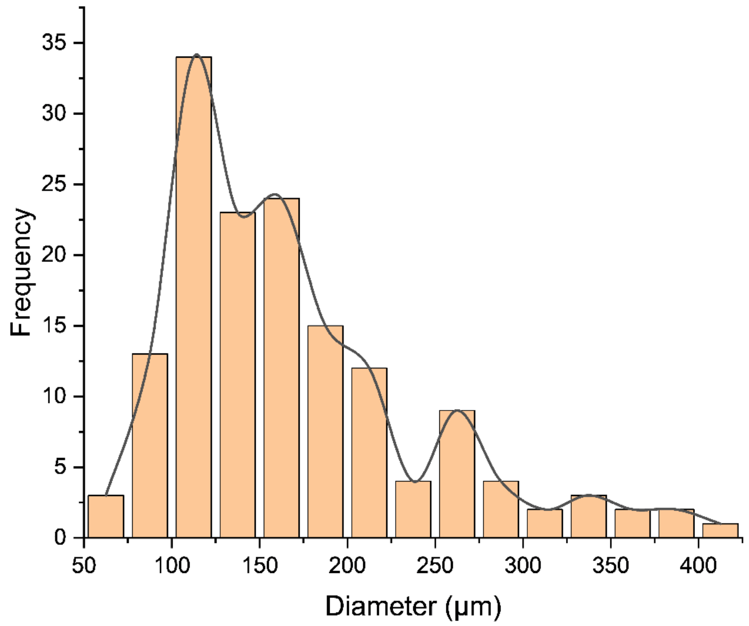


**Figure S1.** Particle size distribution of the powder of TP-NM$_{EL100}$ (n=150).

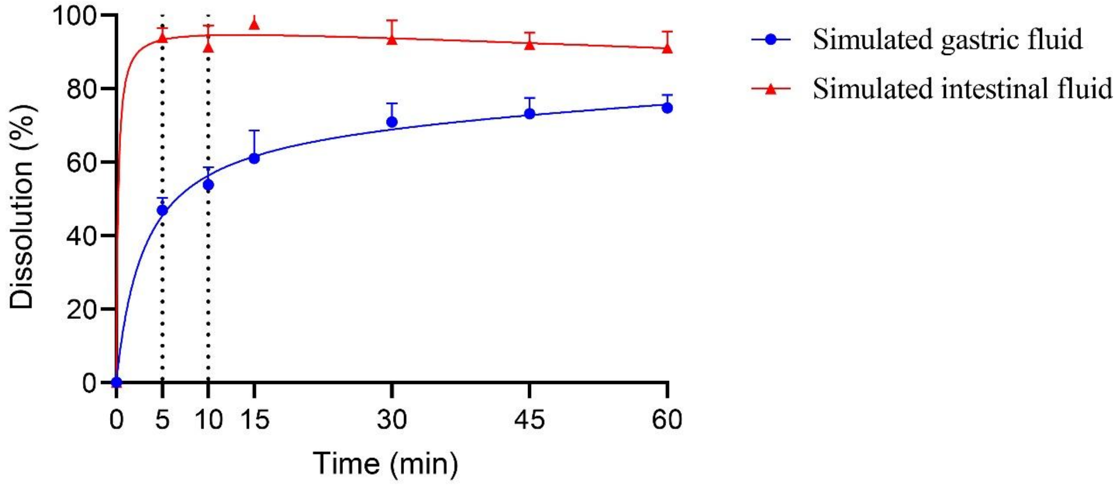


**Figure S2.** *In vitro* release behavior of TP-NM$_{EL100}$ in simulated gastric and intestinal fluids (Mean±SD, n=3).

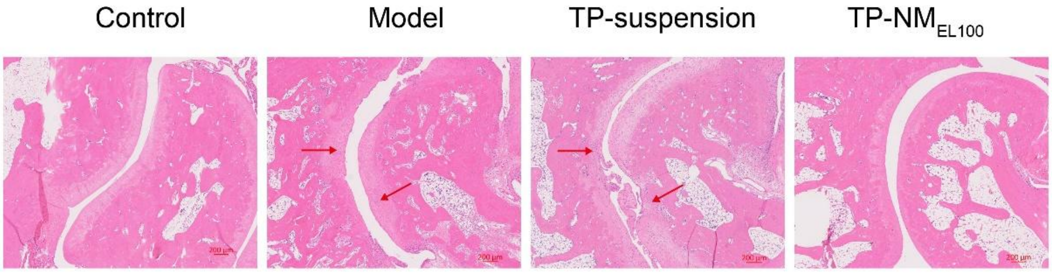


**Figure S3.** Representative H&E-stained joint sections from each group of rats. Red arrows indicate areas of inflammatory cell infiltration.

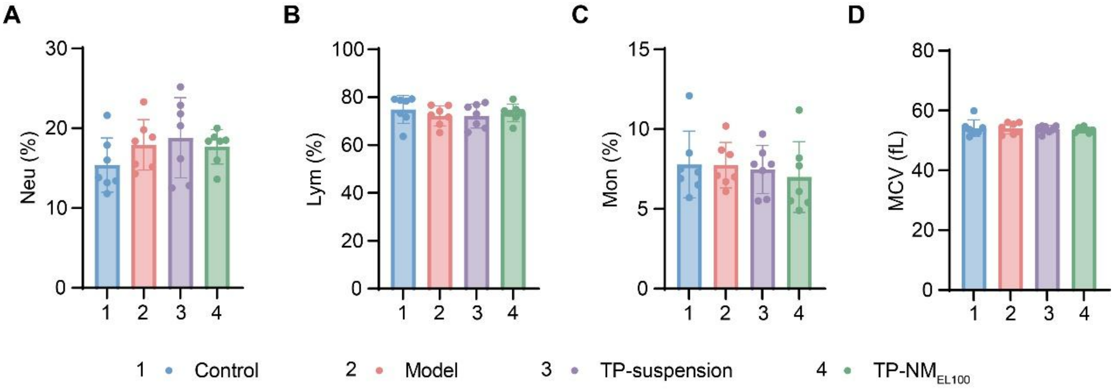


**Figure S4.** Four additional parameters of complete blood count analysis. (A) neutrophil percentage (Neu%), (B) lymphocyte percentage (Lym%), (C) monocyte percentage (Mon%), and (D) mean corpuscular volume (MCV, fL) (Mean±SD, n=7).
